# Supplementary material for: Automated model building and protein identification in cryo-EM maps
Source: bioRxiv. 2023 May 16:2023.05.16.541002. Preprint. [Version 1] doi: 10.1101/2023.05.16.541002 (PMC10245678; doi:10.1101/2023.05.16.541002)
Supplement: Supplementary file 1 [file media-1.pdf]

Automated model building and protein identification in cryo-EM maps

Kiarash Jamali [1*], Lukas Käll [2], Rui Zhang [3],
Alan Brown [4], Dari Kimanius [1*] & Sjors H.W. Scheres [1*]

[1] MRC Laboratory of Molecular Biology,
Cambridge, UK
[2] Science for Life Laboratory, KTH Royal Institute of Technology,
Stockholm, Sweden
[3] Washington University in St. Louis,
St. Louis, MO, USA
[4] Blavatnik Institute, Harvard Medical School,
Boston, MA, USA
[*] Correspondence to {kjamali,dari,scheres}@mrc-lmb.cam.ac.uk

Supplementary information

Changes in ModelAngelo 1.0 We previously described an early (beta) version of ModelAngelo (*1*). Here, we introduce the first stable release of ModelAngelo (version 1.0), which extends the beta version by adding the ability to build nucleotides and an updated Hidden Markov Model algorithm, as described in the main text. We also made minor changes in the graph neural network to improve the performance of ModelAngelo due to the enhanced requirements of building nucleotides. Whereas the beta version of ModelAngelo used cryo-EM maps to a maximum spatial frequency of 3 Å, ModelAngelo 1.0 uses information up to 2 Å resolution. To capture the same context radius, the regions that are sampled around each residue in the cryo-EM module were thus increased from 17 to 23 voxels for the cubes and from 5 to 7 voxels for

the rectangle lengths. We improved the training of the model by using the Lion optimizer (*2*) and changing the dropout probability to 0.1 from 0.5 (*3*). To compensate for the increased computational costs of these changes, we also implemented several approaches to speed up calculations. In particular, ModelAngelo can now be run using multiple GPUs simultaneously, node updates are performed more efficiently, and we use larger batch sizes in training. Furthermore, we confirmed that half-precision inference (running the model with a two-byte floating point precision rather than the default four-byte one) does not affect the outcome in the GNN. As a result of these changes, ModelAngelo 1.0 runs faster than the beta version, even though it uses a larger network.

Identification of radial spoke and central apparatus proteins The structure of radial spoke 1 (RS1) from *Chlamydomonas reinhardtii* (EMD-22475) (*4*) contained unassigned proteins that were either left unmodeled or tentatively interpreted with a poly(UNK) model. To identify these proteins, we ran ModelAngelo without using its sequence module to calculate an initial atomic model with HMM profiles for all chains. We subsequently searched the HMM profiles against the latest version of the *C. reinhardtii* genome (*5*), which was not available at the time of the original publication. For a known radial spoke protein, RSP6, ModelAngelo correctly predicted 67% of all residues even without knowledge of its sequence. This approach also unambiguously identified three unassigned proteins: FAP109, Cre17.g802036, and Cre05.g240450, which we reassign as RSP25, RSP26, and RSP27, respectively. RSP27 was identified from a fragment of just 33 residues, demonstrating ModelAngelo's ability to identify proteins from minimal information, given well-resolved sidechain densities.

RSP25 and RSP26 form a heterodimer in the neck of RS1. These structurally similar proteins each have an N-terminal RIIa domain (similar to the dimerization-anchoring domain of cAMP-dependent protein kinase regulatory subunit) followed by two C-terminal EF-hand mo-

tifs. The proteins were identified based on sequence differences between their better-resolved RIIa domains, demonstrating ModelAngelo's ability to distinguish between similar proteins. RSP25 (FAP109) had been detected by mass spectrometry analysis of RS1 purified from *C. reinhardtii* axonemes (*4*), providing confidence to the assignment. RSP26 (Cre17.g802036) was not annotated in earlier versions of the *C. reinhardtii* genome, explaining its absence from proteomic studies. RSP27 (Cre05.g240450) forms a small, L-shaped helix in the center of the RS1 stalk.

Following identification, we constructed an input sequence file that included all chains in the deposited model along with the three newly identified chains and ran ModelAngelo again. This approach identified and built extensions of RSP16 that had been left unassigned in the deposited model. We then extended the models of RSP25 and RSP26 using AlphaFold2 predictions for the EF-hand motifs which have relatively poor cryo-EM density, demonstrating how ModelAngelo and AI-based structure prediction methods can be used together to build more complete atomic models.

The microtubule-bound stalk of radial spoke 2 (RS2), which is structurally and compositionally different from RS1, also contained unassigned proteins in the deposited map (EMD-22481) (*4*). We therefore applied the same process to identify one additional protein, Cre08.g800895, which we rename as RSP24. RSP24 is a 25 kDa bilobal protein with an N-terminal ubiquitin-like domain. An LC8-interacting protein in the stalk of RS2 remains unassigned due to too few resolved sidechains.

In the axoneme, radial spokes interact transiently with the central apparatus (CA). Structures of the two microtubules (C1 and C2) that together form the *C. reinhardtii* CA have recently become available (EMD-25381 and EMD-25361) (*6*). The map of the C1 microtubule (EMD-25381) contained a number of unassigned densities. We, therefore, applied ModelAngelo without using its sequence module to a `Phenix.auto_sharpened` version of the map,

as the original map was postprocessed with DeepEMhancer (*7*). This approach identified two new proteins: FAP92 and FAP374. FAP92 is a microtubule-associated protein that binds in the inter-protofilament cleft between protofilaments 3 and 4 and repeats with 32-nm periodicity, whereas FAP374 is a microtubule inner protein that repeats with 16-nm periodicity. Neither protein has a globular fold nor is fully resolved in the map, demonstrating ModelAngelo's ability to identify ordered fragments of proteins. The final models of FAP92 and FAP374 were extended manually using Coot (*8*) through regions of less well-resolved density and refined in Phenix (*9*).

References

1. Jamali, K., Kimanius, D. & Scheres, S. H. A graph neural network approach to automated model building in cryo-EM maps. In *The Eleventh International Conference on Learning Representations* (2023). URL `https://openreview.net/forum?id=65XDF_nwI61`.

2. Chen, X. *et al.* Symbolic discovery of optimization algorithms (2023). `2302.06675`.

3. Srivastava, N., Hinton, G., Krizhevsky, A., Sutskever, I. & Salakhutdinov, R. Dropout: a simple way to prevent neural networks from overfitting. *The journal of machine learning research* **15**, 1929–1958 (2014).

4. Gui, M. *et al.* Structures of radial spokes and associated complexes important for ciliary motility. *Nature structural & molecular biology* **28**, 29–37 (2021).

5. Craig, R. J. *et al.* The chlamydomonas genome project, version 6: Reference assemblies for mating-type plus and minus strains reveal extensive structural mutation in the laboratory. *The Plant Cell* **35**, 644–672 (2023).

6. Gui, M., Wang, X., Dutcher, S. K., Brown, A. & Zhang, R. Ciliary central apparatus structure reveals mechanisms of microtubule patterning. *Nature Structural & Molecular Biology* **29**, 483–492 (2022).

7. Sanchez-Garcia, R. *et al.* Deepemhancer: a deep learning solution for cryo-em volume post-processing. *Communications biology* **4**, 874 (2021).

8. Emsley, P., Lohkamp, B., Scott, W. G. & Cowtan, K. Features and development of coot. *Acta Crystallographica Section D - Biological Crystallography* **66**, 486–501 (2010).

9. Afonine, P. V. *et al.* Real-space refinement in phenix for cryo-em and crystallography. *Acta Crystallographica Section D: Structural Biology* **74**, 531–544 (2018).
